# Overview of the epidemiological conditions of HIV among key populations in Africa

**DOI:** 10.1002/jia2.25716

**Published:** 2021-06-30

**Authors:** Harry Jin, Arjee Restar, Chris Beyrer

**Affiliations:** ^1^ Department of Epidemiology Johns Hopkins Bloomberg School of Public Health

**Keywords:** HIV, incidence, surveillance, key populations, Africa, sexual and gender minorities, sex workers, people who inject drugs

## Abstract

**Introduction:**

Despite extraordinary progress in HIV treatment coverage and expanding access to HIV prevention services and that multiple African countries are on track in their efforts to reach 90‐90‐90 goals, the epidemic continues to persist, with prevalence and incidence rates too high in some parts of the continent to achieve epidemic control. While data sources are improving, and research studies on key populations in specific contexts have improved, work on understanding the HIV burdens and barriers to services for these populations remains sparse, uneven and absent altogether in multiple settings. More data have become available in the last several years, and data published in 2010 or more recently are reviewed here for each key population. This scoping review assesses the current epidemiology of HIV among key populations in Africa and the social and political environments that contribute to the epidemic, both of which suggest that without significant policy reform, these epidemics will likely continue.

**Results and discussion:**

Across Africa, the HIV epidemic is most severe among key populations including women and men who sell or trade sex, men who have sex with men, people who inject drugs, transgender women who have sex with men and prisoners and detainees. These groups account for the majority of new infections in West and Central Africa, and an estimated 25% of new infections in East and Southern Africa, despite representing relatively small proportions of those populations. The HIV literature in Africa emphasizes that despite significant health needs, key populations experience barriers to accessing services within the healthcare and legal justice systems. Current shortcomings of surveillance systems in enumerating key populations impact the way funding mechanisms and resources are allocated and distributed. Adapting more equitable and epidemiologically sound frameworks will be necessary for current and future HIV programming investments.

**Conclusions:**

Through this review, the available literature on HIV epidemiology among key populations in Africa brings to light a number of surveillance, programmatic and research gaps. For many communities, interventions targeting the health and security conditions continue to be minimal. Compelling evidence suggests that sweeping policy and programmatic changes are needed to effectively tackle the persistent HIV epidemic in Africa.

## 1 INTRODUCTION

Despite extraordinary progress in HIV treatment coverage and in expanding access to HIV prevention services, the epidemics of West, Central, East and Southern Africa continue in 2020, with incidence rates too high to achieve epidemic control in many countries and populations [1]. Across Africa, prevalence burdens and incidence rates remain highest among “key populations” including women and men who sell or trade sex, men who have sex with men (MSM), people who inject drugs (PWID), transgender women who have sex with men and prisoners and detainees [1, 2, 3, 4, 5, 6, 7, 8, 9, 10]. These groups, often clustered together as key populations, account for the large majority of new infections in West, North and Central Africa, and an estimated 25% of new infections in East and Southern Africa, despite representing relatively small proportions of those overall populations [1]. Attributable fraction analyses of female sex workers (FSW) in South Africa, where some 70% of these women were living with HIV in 2018, demonstrate that infections in these women also contribute significantly to incident infections in men and overall epidemic trajectories [11]. Yet these individuals and their communities remain marginalized in the HIV response, criminalized in too many settings and excluded from essential services across the continent. This has been true for decades, and the subject of much HIV advocacy, human rights concern and public health intervention. Yet few governments have been willing or able to make significant policy progress in addressing the structural changes needed to change these realities on the ground. This has led to the ongoing spread of HIV infection despite treatment gains and the perpetuation of HIV sub‐epidemics, and at least in some cases, the inability to achieve wider control.

Progress towards improving HIV outcomes for key populations across Africa was insufficient before the impact of COVID‐19, and early evidence suggests the new pandemic has had significant impacts on both HIV testing and HIV prevention services [12], complicating the epidemiological picture and potentially impacting what declines in incidence that may have been underway. This scoping review will assess the evidence on prevalence and incidence, where it is available, among key populations in Africa. Based on these findings, we then offer recommendations for significant policy reform in order to address these epidemics.

While data sources are improving, and empirical studies on key populations in specific contexts have improved, work on understanding the HIV burdens and barriers to services for these populations remains sparse, uneven and sometimes absent altogether in multiple settings. The criminalization of the same‐sex behaviour in more than half of the countries across the continent [13], for example, makes an assessment of the HIV epidemic and its trajectory among gay, bisexual and other MSM, challenging and even risky in some settings. Political factors have played oversized roles in many settings, as was seen in Tanzania when a regime change led to the closure of some 30 clinics nationwide which had been providing services to sex workers and sexual and gender minority clients [14].

## 2 METHODS

We conducted a scoping review of recently published articles reporting the epidemiology of HIV among key populations across Africa, including MSM, men who sell/trade sex, women who sell/trade sex, transgender women, PWID and prisoners and detainees [15]. Peer‐reviewed papers and grey literature that reported on the incidence and/or prevalence of HIV among key populations were identified by our research team between 13 July 2020 and 12 October 2020. Scoping reviews do not use a priori article criteria, however, we focused on papers published in 2010 or more recently, from Africa, and which included HIV prevalence, incidence or both, among the key populations under study.

Specifically, this review included articles that reported HIV prevalence estimates and incidence rates at the country and community levels. We searched through grey literature and keyword searches in PubMed for the following terms: Africa, HIV, HIV prevalence, HIV incidence, MSM, male sex workers (MSW), men who sell sex, men who trade sex, FSW, women who sell sex, women who trade sex, transgender women, PWID, prisoners and detainees and different iterations of these terms. We also identified studies in the articles’ reference lists. The geographical reach of this effort includes some North African countries since HIV epidemics in this region have been expanding, and since HIV spread in this region has been significant among PWID and sexual and gender minorities. Our team screened abstracts to identify potential studies that would be pertinent to this review, and papers that passed our initial screening were assessed to ensure that the research methodologies were rigorous. This scoping review provides a summary of recently published literature that characterizes HIV epidemiology among key populations in Africa. Additionally, this manuscript also highlights the gaps in literature and research that hinder policy and programme development focused on improving the well‐being of key populations.

## 3 RESULTS AND DISCUSSION

### 3.1 African MSM

MSM are disproportionately burdened by HIV globally, however, HIV research among MSM in Africa had been neglected until the past two decades [4, 16]. The lack of MSM‐focused surveillance, programmes and research may partially be explained by heterosexual and vertical transmission historically accounting for the majority of HIV infections in Africa, however, lesbian, gay, bisexual, and transgender (LGBT)‐related stigma and discriminatory laws that are pervasive across Africa not only discourage MSM from disclosing same‐sex behaviours, but also prevent the prioritization of MSM‐focused research by government bodies [17]. Despite these challenges, country‐level surveillance studies have reported wide ranges of HIV prevalence estimates across and within countries, highlighting the challenge of characterizing the HIV epidemic among MSM across the continent.

The 2020 UNAIDS Global AIDS Update provided HIV prevalence estimates from 2019 among MSM for 16 of the 54 countries in Africa (Table 1), ranging from 0% in Comoros to 23.4% in Mauritania. Although some country‐level HIV prevalence estimates specific to MSM are comparable to that for the general population [1], population‐based studies, particularly ones conducted in areas in which MSM are concentrated, have demonstrated high relative prevalence burdens of HIV among these men [18, 19, 20]. A meta‐analysis of 17 studies reported an average prevalence of 17.8% (range: 3.7% ‐ 33.4%) among MSM in Africa, nearly five times higher than the prevalence among men in the general population [2]. However, the average HIV testing rate among MSM in Africa is suboptimal [21], suggesting that what is reported in studies underestimates the true prevalence rate [20]. Furthermore, the proportion of HIV‐positive MSM in Africa who use ART and are virally suppressed remains low (24% and 25% respectively) [21], which will continue fuelling the epidemic in this population.

**Table 1:** 2020 UNAIDS Global AIDS Update HIV prevalence estimates among key populations in African countries

	General population, %	Men who have sex with men, %	Women who sell/trade sex, %	Transgender women	People who inject drugs, %	Prisoners and detainees, %
Angola	1.9	2.0	8.0			15.9
Benin	1.0	7.0	8.5		2.2	0.6
Burkina Faso	0.7	1.9	5.4			2.2
Cameroon	3.1	20.6	24.3			4.0
Comoros	<0.1	0	0.3		1.8	
Cote D’Ivoire	2.4	12.3	7.5		3.4	1.2
Djibouti	0.8	14.2	9.3			
Egypt	<0.1	6.7	2.8		2.5	
Eritrea	0.6					1.4
Lesotho	22.8					31.4
Malawi	8.9	6.8	55.0			19.0
Mali	1.2	12.6	8.7	11.7		1.6
Mauritania	0.2	23.4	9.0			2.9
Seychelles			4.6		23.0	9.9
Sierra Leone	1.6	14.0	6.7	15.3	8.5	8.7
South Sudan	2.5		11.4			
Sudan	0.2	0.8	0.7			
Tanzania	4.8	8.4	15.4			6.7
Togo	2.2	22.0	13.2			
Tunisia	<0.1		1.2		6.0	
Uganda	5.8		31.3		17.0	4.0
Zambia	11.5		48.8			27.4
Zimbabwe	12.8	21.1	42.2			28.0

Anti‐LGBT public sentiment and legislation in Africa have dire health implications for MSM, especially with respect to HIV care and prevention [22]. LGBT‐ and HIV‐related stigma are known contributors to the HIV epidemic in Africa, impacting every step of the HIV care continuum. Consistent with other studies, research conducted among African MSM have demonstrated that enacted and perceived stigma prevent MSM from seeking HIV testing [23], accessing HIV care [24], being retained in care [25], obtaining ART [25] and achieving viral suppression [26]. There are also documented cases in which healthcare providers have stopped providing services to MSM over the fear of harassment [27]. Punitive laws that criminalize homosexuality, such as the Same‐Sex Marriage Prohibition Act in Nigeria, violate human rights statutes and reinforce the culture of fear that marginalize MSM, globally [17]. A seminal study examining the effects resulting from the enactment of the Same‐Sex Marriage Prohibition Act reported that MSM were not only less likely to access healthcare due to fear of discrimination following the passing of the legislation but also subject to more instances of abuse [25], perpetuating the disenfranchisement of the MSM community.

### 3.2 Men who sell/trade sex

The growing body of literature characterizing the epidemiology of HIV among MSM in Africa consistently reports a high prevalence of HIV in addition to an increased risk of HIV transmission [3, 4]. An understudied population which is often considered to be nested within the greater MSM population are MSW, who have been identified to be at even greater risk of HIV infection compared to the general MSM population [28, 29]. The elevated HIV risk among MSW is often attributed to the occupation‐related factors associated with sex work (e.g. financial incentivization of condomless anal sex, higher risk of acquiring sexually transmitted infections (STIs), multiple partners) compounded with the factors that influence HIV risk among MSM [30].

While the literature describing the epidemic among MSW in Africa is limited, recent studies have demonstrated a high burden of HIV. For example, a cross‐sectional study of MSM in Nairobi, Kenya, reported that the HIV prevalence among MSW in their sample was 26.3% compared to 12.2% among the MSM who did not sell sex [31]. Similarly, a prospective cohort study of MSW in Nairobi reported a baseline HIV prevalence of 40% (203/507) and an incidence rate of 10.9 infections per 100 person‐years [32], whereas the HIV prevalence and cumulative incidence among the general Kenyan adult population are estimated to be 4.5% and 0.92 infections per 1000 persons [1, 33].

Improving surveillance methods to characterize the epidemiology of HIV among MSW remains a crucial and challenging task. In addition to the dearth of research focusing on this population in Africa, the varying levels of quality with respect to methodological rigour employed in research studies impede our ability to make meaningful comparisons between regions and/or trends over time [34]. Additionally, the quality of data collection and sampling may also complicate comparisons. For example, there are notable discrepancies between data reported to the United Nations General Assembly (UNGA) and the statistics reported in peer‐reviewed journal articles. UNGA collected HIV prevalence data among MSW from 52 countries in 2012. The median HIV prevalence among the five African countries that reported data was 12.5%. In contrast, HIV prevalence estimates among MSW in Africa reported in peer‐reviewed journal articles were markedly higher, with estimates ranged from 19.7% in Mombasa, Kenya [35], to 50.0% in Abidjan, Cote D’Ivoire [36]. While it is challenging to definitively assess which figures are more reflective of the true prevalence among these men, differences in definitions, sampling methodologies and the ability to engage these men and their communities in research, almost certainly contribute to these variations in measurement of HIV burden.

A recent review that summarized the practices, contexts and HIV risk among MSW presented recommendations to improve the quality of MSW‐specific data collection and reporting. These recommendations included clarifying the definition for what constitutes sex work, differentiating between lifetime sex work and current sex work, refining risk transmission categories to delineate between populations with intersecting risk behaviours, and collecting comprehensive and granular data regarding career duration and number of paid acts to examine the potential dose–response relationship between sex work and HIV transmission risk [34].

### 3.3 Women who sell/trade sex

FSW are among the most at risk populations for HIV/AIDS in Africa, with an HIV prevalence reaching nearly 40% [5]. Country‐level HIV prevalence estimates among FSW presented in the 2020 UNAIDS Global AIDS Update were available for 21 countries, of which eight were over 10% (Table 1); Malawi 55.0%; Zambia 48.8%; Zimbabwe 42.2%; Uganda 31.3%; Cameroon 24.3%; Tanzania 15.4%; Togo 13.2%; South Sudan 11.4%) [1].

In addition to the occupational hazards associated with sex work that elevate HIV transmission (e.g. multiple sexual partners, condomless sex, untreated STI), the high HIV prevalence among FSW in Africa is also heavily influenced by legal frameworks on commercial sex work [37]. The relationship between HIV risk and the legal status of sex work has been well demonstrated [38, 39], and results from mathematical models suggest that the decriminalization of sex work may significantly reduce HIV incidence [40]. Despite compelling evidence that emphasizes the need for legal reform, no countries have decriminalized sex work in the past five years [41, 42]. Furthermore, FSW in Africa have little to no recourse to report violence or abuse, including police violence, which reduces their ability to negotiate condom use and leaves them vulnerable to rape [43, 44].

Punitive laws not only increase vulnerabilities among sex workers (e.g. unregulated work environments, increased economic insecurities), but also exacerbate sex work‐related stigma, which decreases engagement in HIV prevention and treatment services [38, 45]. Expansion of HIV prevention and treatment services in Africa has led to substantial improvements in HIV‐related health outcomes among the general population, however, FSW have not equitably benefitted from efforts to increase HIV service coverage [46]. FSW who have sought HIV prevention and treatment services have reported gender‐based violence, police harassment and discrimination from healthcare providers, all of which deter FSW from accessing the care they require [46, 47, 48]. Some African states have prioritized women selling or trading sex for PrEP programmes, including Kenya and South Africa, though uptake, retention and persistence on daily oral pre‐exposure prophylaxis (PrEP) have remained challenging.

### 3.4 Transgender women

Globally, including in multiple African countries, communities of transgender (trans) women who have sex with men are becoming more acknowledged as a priority population for HIV prevention and care [49]. Emerging evidence documents high HIV infection rates among this population, with pooled estimated global HIV prevalence of 19% [6]. In Africa, a handful of multi‐country community‐based sampling studies have reported HIV prevalence ranging from 25.0% in one study (n = 235/926) [50], which surveyed countries of Burkina Faso, Cote d’Ivoire, The Gambia, Lesotho, Malawi, Senegal, Swaziland and Togo to 42.6% in another study (n = 58/136) [51], which surveyed countries of Kenya, Malawi and South Africa. Studies investigating HIV incidence rates also vary from 4.7 infections per 100 person‐years in South Africa [52] to as high as 20.6 infections per 100 person‐years in Kenya [53].

Current literature on HIV among trans women in Africa documents various socio‐ecological (i.e. behavioural, social and structural) factors that place them at increased risk for HIV. In particular, recent studies document trans women reporting greater levels of sex work engagement with cisgender men, partaking in condomless receptive anal intercourse, experiencing sexual behaviour stigma from family and peers and being excluded from their biological families [50, 51, 54]. Notably, in one study [50], trans women reported higher levels of depressive symptoms (57.3%, n = 536/937) and intimate partner violence like forced sex (26.7%, n = 250/937), and also found that structural stigma, violence and depression were associated with HIV. These findings are aligned with other HIV studies theorizing syndemic impact of various socio‐ecological factors on HIV among trans women [55, 56].

Designing studies specific to addressing multiple socio‐ecological drivers of HIV among African trans populations remain a challenge. Samples of trans women in the continent have relied on sub‐analysing studies that were mainly designed for recruiting cisgender MSM, employing trans women as ancillary samples to this population [57]. This remains problematic providing that studies showing that trans women have distinct differences in both HIV‐related experiences and behaviours from cis‐MSM [54], and that researchers and activists have called for prioritization of developing HIV interventions specific to trans women. Moreover, while the literature on African trans women is scant, there is also a dearth of research in HIV prevention and care among the larger communities of trans and non‐binary people in Africa. Given that trans communities are non‐monolithic, there is a need to bolster research and characterize HIV prevention and treatment continuum outcomes across trans and non‐binary populations and across the great diversity of Africa’s cultures, languages and ethnicities. Future HIV research among trans populations in Africa should address these research pitfalls and must employ current best practices centred on gender affirmation models.

### 3.5 People who inject drugs

Country‐level HIV prevalence estimates among PWID presented in the 2020 UNAIDS Global AIDS Update were available for eight countries, ranging greatly across and within regions of Africa [1]. Prevalence estimates ranged from 1.8% (Comoros) to 23.0% (Seychelles) in East and Southern Africa and 2.2% (Benin) to 8.5% (Sierra Leone) in West and Central Africa (Table 1). PWID accounted for approximately 43% of new HIV infections in North Africa, however, country‐level data were only available for Egypt (2.5%) and Tunisia (6.0%).

A 2014 systematic review of articles reporting HIV prevalence estimates among PWID in the Middle East and North Africa identified studies conducted in Egypt, Libya and Morocco [58]. While the range of prevalence estimates reported by studies that were conducted in Egypt were similar to what was reported in the 2020 UNAIDS Global AIDS Update, the authors of the review identified one study which estimated an HIV prevalence of 87% among PWID in Libya [59]. Heterogeneity in the prevalence of HIV among PWID within countries is common across Africa [60, 61, 62] A study across six states in Nigeria reported HIV prevalence estimates ranging from 2.4% to 9.3% [61]. Similarly, studies conducted across South Africa and Kenya reported estimates ranging from 9% to 17% and 14.5% to 20.5% respectively [60, 62]. The scarcity of country‐level HIV data in addition to the wide prevalence ranges within regions among PWID in Africa highlights the importance of comprehensive coverage of routine HIV surveillance programmes.

Harm reduction services, substance use treatment programmes and HIV services targeting PWID are not widely available across Africa. However, there has been a concerted effort to implement comprehensive interventions, including programmes that incorporate HIV prevention and treatment [63]. Methadone has been introduced in several African countries with large PWID populations (e.g. Tanzania, Kenya), and research has demonstrated that methadone treatment programmes serve as an effective mechanism through which HIV prevention, treatment and care can be delivered to improve the health of PWID [64, 65, 66]. Additionally, law enforcement has become recognized as a critical agent in diverting PWID from the criminal justice system to health and social services. Compelling evidence supporting the efficacy of integrating substance use and HIV services have resulted in the gradual adoption of programmes, such as HIV prevention interventions specifically tailored for PWID, addiction treatment and community outreach across the region [63, 67, 68].

### 3.6 Prisoners and detainees

The high prevalence of HIV in prisons is a global public health issue [69]. While cases of HIV transmission within prison settings remain uncommon, populations at high risk for HIV infection, particularly PWID, are overrepresented in prisons [70]. Although prisons may be conducive environments for infectious diseases to spread, they can also provide opportunities to treat and prevent further infections [71, 72].

The UNAIDS 2020 Global AIDS Update reported country‐level HIV prevalence estimates among prisoners in 15 countries across Africa (Table 1) [1]. The estimates ranged from 1.4% (Eritrea) to 31.4% (Lesotho) in East and Southern Africa and 0.6% (Benin) to 8.7% (Sierra Leone) in West and Central Africa.

The HIV prevalence estimates reported in peer‐reviewed academic journals consistently reported higher prevalence estimates relative to what was reported in the UNAIDS report. A systematic review published in the Lancet in 2016 reported prevalence estimates ranging from 4.2% (Ethiopia) to 23.0% (Malawi) in East Africa, 7.2% (South Africa) to 34.9% (Swaziland) in Southern Africa and 2.3% (Ghana) to 10.2% (Congo) in West and Central Africa [7] Additionally, a cross‐sectional study of five correctional facilities across South Africa estimated that the HIV prevalence among prisoners was 17.7% (95% CI = 17.2% to 18.3%) [73].

Recent data describing the HIV epidemic among prisoners in North Africa are sparse. There were notably no data describing the HIV prevalence among prisoners in North African countries in the UNAIDS 2020 Global AIDS Update and the World Health Organization’s (WHO’s) 2012 *HIV Surveillance in the WHO Eastern Mediterranean Region: Regional Update* report only provided prevalence estimates among prisoners in two North Africa countries, Egypt (<0.001% in 2009) and Morocco (0.5% among male prisoners in 2010) [74]. One study of 6,371 prisoners in Libya reported an HIV prevalence of 18.2% [75], which was markedly higher than any estimates from this region.

The WHO’s *Focus on Key Populations in National HIV Strategic Plans in the WHO Africa Region* report published in 2018 highlighted that the great majority of the national strategic plans in the WHO African Region (38 of 45) included steps to improve HIV surveillance and/or care among people in prisons and other closed settings [76]. Additionally, about two‐fifths of the national strategic plans included steps to measure HIV prevalence among this population.

### 3.7 Discussion

The HIV literature in Africa emphasizes that despite significant health needs, key populations experience barriers to accessing services within the healthcare and legal justice systems. This review found that LGBT‐, sex work‐, drug‐ and HIV‐related discrimination – and in many cases, the ubiquitous legal landscape across the continent – prevent key populations from accessing services pertinent to each step of the HIV continuum (e.g. HIV testing [23], care linkage and retention [24, 25], antiretroviral therapy uptake and adherence [25]. Key populations experience social and structural barriers to accessing HIV prevention and care services as well as other pertinent primary care routine services due to stigma and discrimination, denial of services and verbal and physical abuse by providers. Recent enforcement of anti‐LGBT laws, such as the Same‐Sex Marriage Prohibition Act, shows that these barriers further elevate challenges in accessing HIV services for MSM and transgender communities [17]. Moreover, the stigma and criminalization of substance use, sex work and same‐sex practices contributes to negative interactions between police and key populations, and in particular MSM, transgender, sex worker and PWID communities. These interactions include arbitrary arrests, violence and sexual abuse of key populations by law enforcement officers [77]. Such negative interactions place key populations at the margins of healthcare and legal justice systems – driving them further away from attaining services that promote their legal protection, health and well‐being. As such, the need to align social and political climate, including provider and law enforcement officer’s attitudes towards key populations, with public health efforts to improve HIV‐related outcomes of key populations in Africa remains pertinent.

This review also highlighted the need to tailor and expand the integration of HIV programming with other life‐saving services including harm reduction, gender‐affirmation, gender‐based violence prevention and support services. While such services are not currently widely available across the continent, it is critical for HIV programming to strive to address and meet the co‐occurring health needs of key populations such as PWID and transgender women holistically that also compounds the risk for HIV acquisition and poorer HIV‐related outcomes [49]. Programmes such as community‐based needle and syringe programmes, as well as expansion of current national HIV strategic plans like in South Africa to include recognition of transgender women’s gender affirmation needs within HIV prevention and care services offer promising integrated steps to address gender‐based and/or drug‐related HIV epidemics [49, 63]. The outreach and involvement of key populations in the implementation of these programmes, when proven effective, will be critical to its scale‐up success.

In alignment with numerous community activists and researchers working to improve HIV outcomes in the continent [49, 78], these results also point to the issues surrounding population size estimates and improving HIV epidemiological surveillance systems through transformative changes [79]. Given the current shortcomings of surveillance systems in enumerating key populations accurately as well as the current social and political climate that provides a challenging environment for surveillance systems to reach key populations effectively, current population estimates are likely an underestimation, particularly those reported to UNAIDS [80]. For example current HIV surveillance system remains gender non‐inclusive [79], that is, it currently only defines and recognizes binary cisgender (or non‐transgender) identities in its data collection – a point for future improvement. Additionally, while current tools for estimating population size [81, 82, 83, 84] (e.g. capture‐recapture, network scale‐up, mapping and enumeration, service and unique object multiplier, wisdom of the crowd, estimation using demographic methods) all have common sources of biases and limitations, and that estimates provided from each tool vary in the same population, it is critical to note that the current political and social climate continue to erase and drive key populations across the continent away from being counted – and in turn, continue to undermine the accuracy of these methods [83, 85]. For example, in a 2016 study conducted in Nairobi [82], using wisdom of the crowd method provided a population size estimates of 3,000 PWID and 10,000 FSW, whereas another method using literature and demographic data yielded 6,562 PWID and 29,494 FSW population size estimates, revealing the variability in estimates provided using different tools but estimating the same population. Moreover, this review also finds that estimates on care and treatment cascade and other important social and structural drivers of HIV (e.g. punitive laws) exist for some key populations but not others, revealing that such indicators are collected ununiformly across key populations. Given that these estimates continue to play a critical role in the way funding mechanisms and resources are allocated and distributed, adapting more equitable and epidemiologically sound frameworks will be necessary for current and future HIV programming investments. We are unable to understand the scope of the epidemiology among key populations in Africa without reliable population size estimates. Additionally, country‐level estimates published by organizations such as UNAIDS often require the consent of the participating countries’ governments, which may result in a range of potentially biased sources. Transparency with respect to the research that informs these estimates would improve the public’s confidence in these reports. These gaps in knowledge also have important downstream affects that dictate how much funding a country receives from organizations such as the Global Fund. Research conducted to estimate the population size of each key population should be prioritized to allow researchers and funding entities to appropriately allocate commensurate funds to address the HIV epidemic in Africa.

## 4 CONCLUSIONS

Through this review, the available literature on HIV epidemiology among key populations in Africa brings to light several important findings and a number of surveillance, programme and research gaps. A notable lack of HIV studies as well as surveillance data from various regions of Africa, particularly North Africa, was a common theme throughout all included key populations in this review – warranting an improvement in the quality and scope of surveillance systems to understand and address the HIV disparities in the continent. However, it is clear from what literature does exist that key populations included in this review remain disproportionately burdened by HIV. Such disparities are driven, in part, due to the varying social and political climate across regions of the continent that impact uptake of HIV prevention and care services, pointing to the need to address the societal and political factors that shape and place key populations at risk for HIV acquisition and poorer HIV‐related outcomes. This has been true for at least a decade, and yet progress on the policy and political fronts has been modest at best in some countries and has proven enormously difficult in others. For many communities, health and security conditions have arguably worsened [25, 38, 59].

## Competing interests

The authors declare that they have no competing interests.

## Authors’ contributions

HJ, AR and CB conceptualized, wrote, and edited this paper. All authors have read and approved the final manuscript.
